# Whole exome sequencing of independent lung adenocarcinoma, lung squamous cell carcinoma, and malignant peritoneal mesothelioma: A case report

**DOI:** 10.1097/MD.0000000000005447

**Published:** 2016-12-02

**Authors:** Irene Vanni, Simona Coco, Silvia Bonfiglio, Davide Cittaro, Carlo Genova, Federica Biello, Marco Mora, Valeria Rossella, Maria Giovanna Dal Bello, Anna Truini, Barbara Banelli, Dejan Lazarevic, Angela Alama, Erika Rijavec, Giulia Barletta, Francesco Grossi

**Affiliations:** a Lung Cancer Unit, IRCCS AOU San Martino - IST Istituto Nazionale per la Ricerca sul Cancro, Genova; b Centre for Translational Genomics and Bioinformatics, IRCCS San Raffaele Scientific Institute, Milan; c Department of Internal Medicine and Medical Specialties (DIMI), Università di Genova IRCCS AOU San Martino - IST Istituto Nazionale per la Ricerca sul Cancro, Genova; d Department of Pathology, IRCCS AOU San Martino – IST Istituto Nazionale per la Ricerca sul Cancro, Genova; e Laboratory of Tumor Epigenetics, IRCCS AOU San Martino - IST Istituto Nazionale per la Ricerca sul Cancro and Department of Health Sciences, Università di Genova, Genova, Italy.

**Keywords:** clonal origin, mesothelioma, multiple lung cancers, tumor susceptibility, whole exome sequencing

## Abstract

The presence of multiple primary tumors (MPT) in a single patient has been identified with an increasing frequency. A critical issue is to establish if the second tumor represents an independent primary cancer or a metastasis. Therefore, the assessment of MPT clonal origin might help understand the disease behavior and improve the management/prognosis of the patient.

Herein, we report a 73-year-old male smoker who developed 2 primary lung cancers (adenocarcinoma and squamous cell carcinoma) and a malignant peritoneal mesothelioma (PM).

Whole exome sequencing (WES) of the 3 tumors and of germline DNA was performed to determine the clonal origin and identify genetic cancer susceptibility.

Both lung cancers were characterized by a high mutational rate with distinct mutational profiles and activation of tumor-specific pathways. Conversely, the PM harbored a relative low number of genetic variants and a novel mutation in the *WT1* gene that might be involved in the carcinogenesis of nonasbestos-related mesothelioma. Finally, WES of the germinal DNA displayed several single nucleotide polymorphisms in DNA repair genes likely conferring higher cancer susceptibility.

Overall, WES did not disclose any somatic genetic variant shared across the 3 tumors, suggesting their clonal independency; however, the carcinogenic effect of smoke combined with a deficiency in DNA repair genes and the patient advanced age might have been responsible for the MPT development. This case highlights the WES importance to define the clonal origin of MPT and susceptibility to cancer.

## Abstract 2

Supplemental Digital Content is available in the text

## 1 Introduction

The incidence of multiple primary tumors (MPT) during an individual's lifetime is increasing, mainly due to the advent of accurate cancer secondary prevention programs and the increase of life expectancy for cancer patients. The development of multiple primary lung cancers (MPLC) is an uncommon event, although the improvement in the diagnostic tests and novel therapies able to influence survival after the first diagnosis of cancer have led to an incidence peak that has grown up to 20% over the past 10 years.^[1,2]^ A correct understanding whether the second tumor is an independent primary lesion or a metastasis is fundamental for an adequate therapeutic management of these patients. Currently, the main criteria for defining the lineage of multiple unrelated intrapulmonary tumors compared with metastatic lesions are based on pathological and clinical assessments.^[3,4]^ To date, several studies have described MPLC cases,^[1]^ but most of them have analyzed a limited number of genetic markers, resulting in a low accuracy and limited ability to establish cancers clonality.^[5,6]^ Next generation sequencing (NGS) is a recent technology that can contribute to understanding the molecular mechanisms underlying tumor development by screening the whole DNA mutational profile.^[7–10]^ Recently, Murphy et al^[11]^ applied the NGS approach to define the lineage of MPLC, demonstrating how genomic rearrangements were able to distinguish MPLC from metastatic lesions; however, the authors did not evaluate somatic and germinal mutational profiles. Once established that MPLC are primary and independent tumors, understanding the intrinsic genetic susceptibility to develop multiple cancers during the lifetime is crucial; indeed, those subjects with high predisposition might be enrolled in prevention programs and benefit from personalized follow-ups. Herein, we report an interesting case of a patient that developed 2 primary histologically distinct lung tumors and a malignant PM after 6 years. WES allowed us to deeply screen the 3 tumors, in order to identify a mutational signature specific for each malignancy and to establish the clonal origin of cancers. Concomitantly, the sequencing of normal genomic DNA (gDNA) allowed the identification of germline genetic variants potentially correlated with an individual risk of developing multiple cancers.

## 2 Case report

**Figure 1:**
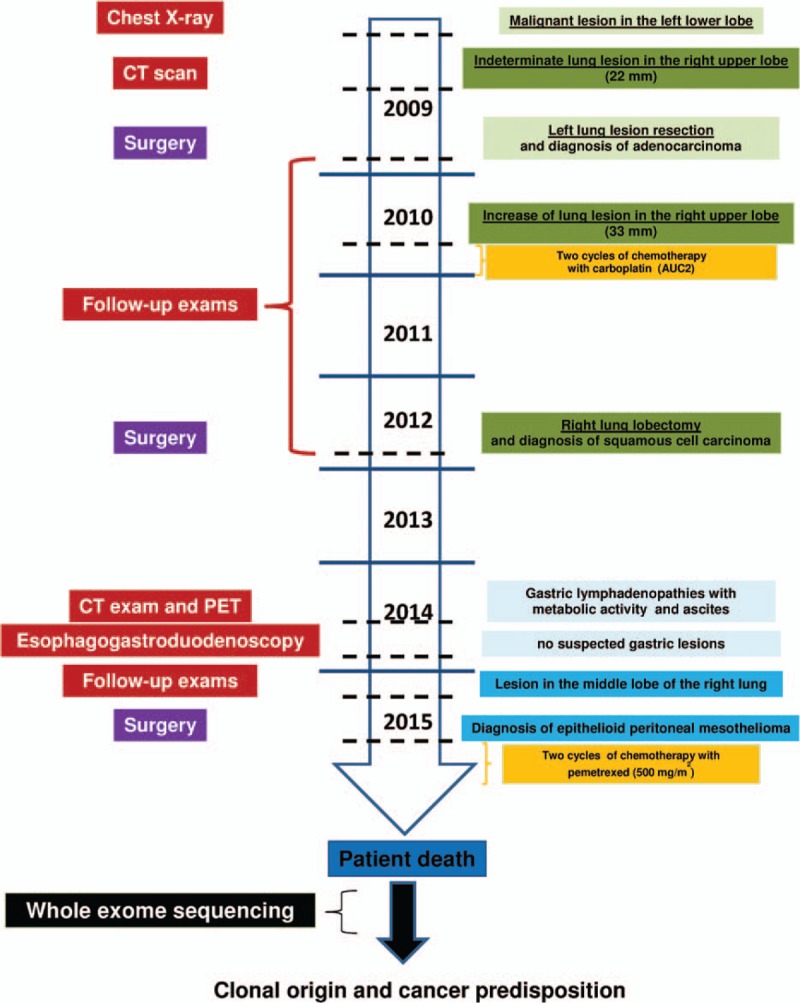
Timeline of oncologic history of the patient. Dashed line means the time of each diagnostic examination (red box) or surgical intervention (violet); Light green, dark green, and dark/light blue boxes report the ADC, SCC, and PM evolution, respectively. Yellow box describes the pharmacological treatment.

**Figure 2:**
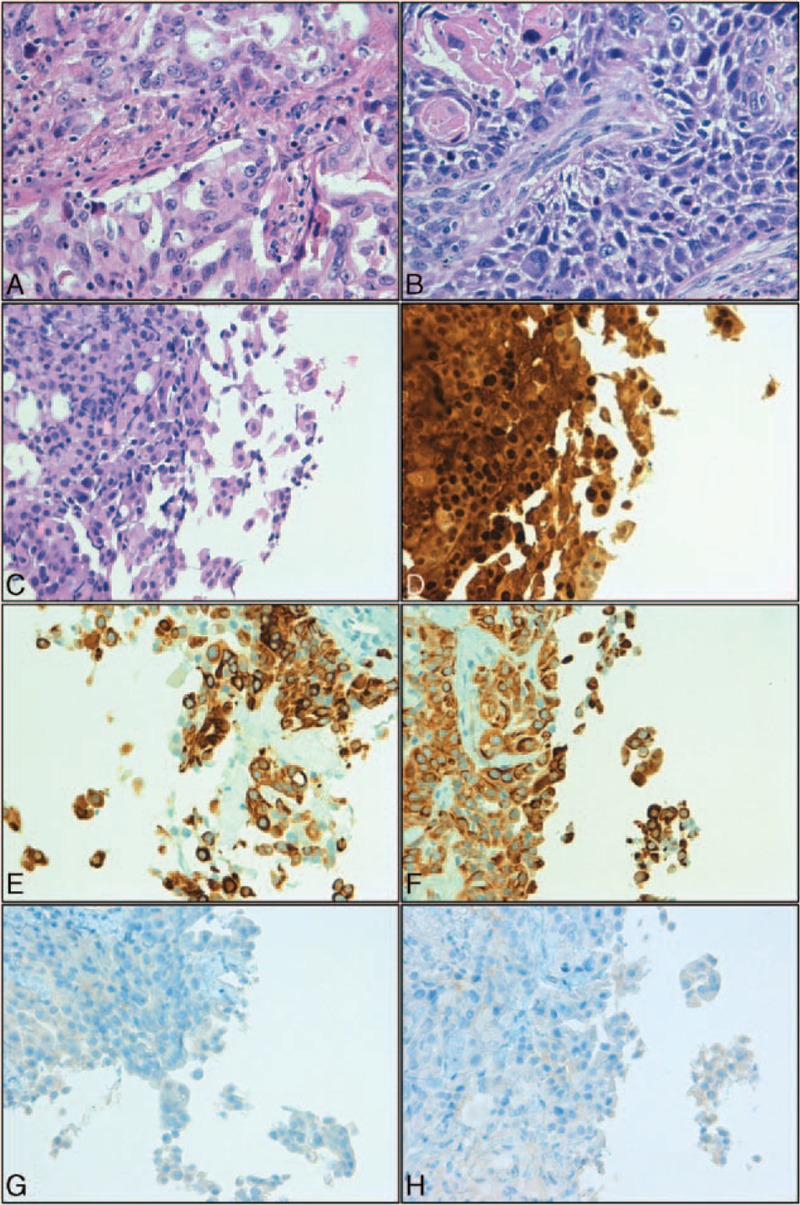
Hematoxylin and eosin stained images of ADC (A), SCC (B), and PM (C) (Original magnification 40x). Immunohistochemistry of PM reported a positive staining for Calretinin (D), CK-7 (E), and CK5&6 (F), whereas a negative staining for TFF-1 (G) and p63 (H) (Original magnification 40x).

We describe the case of a Caucasian male patient with a medical history of heavy smoking habit (100 pack-years), chronic obstructive pulmonary disease (COPD), and no exposure to asbestos. Before being referred to our unit, the patient was initially followed and treated in a different institution; hence, part of the patient's oncologic history was retrospectively retraced when he came to our attention (Fig. 1). In January 2009, the patient, aged 73 years, was subjected to a chest X-ray as preoperative examination for minor surgery with the incidental detection of a suspicious opacity in the left lower lobe. The subsequent diagnostic work-up confirmed a high-risk lesion in the left lower lobe; in addition, the computed tomography (CT) scan identified a smaller lesion in the right upper lobe (22 mm), which was considered an indeterminate lung nodule due to its morphologic characteristics, along with unspecific micro-nodules in the same lung. As the position of the pulmonary findings and the structural lung alterations caused by COPD prevented the collection of bioptic samples, the decision of approaching the left lung lesion with surgery and periodically evaluate the evolution of the indeterminate nodule was taken. Hence, the patient underwent left lung lower lobe segmental resection in April 2009, with postoperative diagnosis of stage IB lung adenocarcinoma (ADC) with solid and glandular patterns and foci of mucus secretion (Fig. 2A). The immunohistochemistry (IHC) analysis revealed positivity for TTF-1, consistently with the diagnosis of a lung primary tumor. The postoperative pathological staging was pT2a, G3, Nx, Mx.

**Figure 3:**
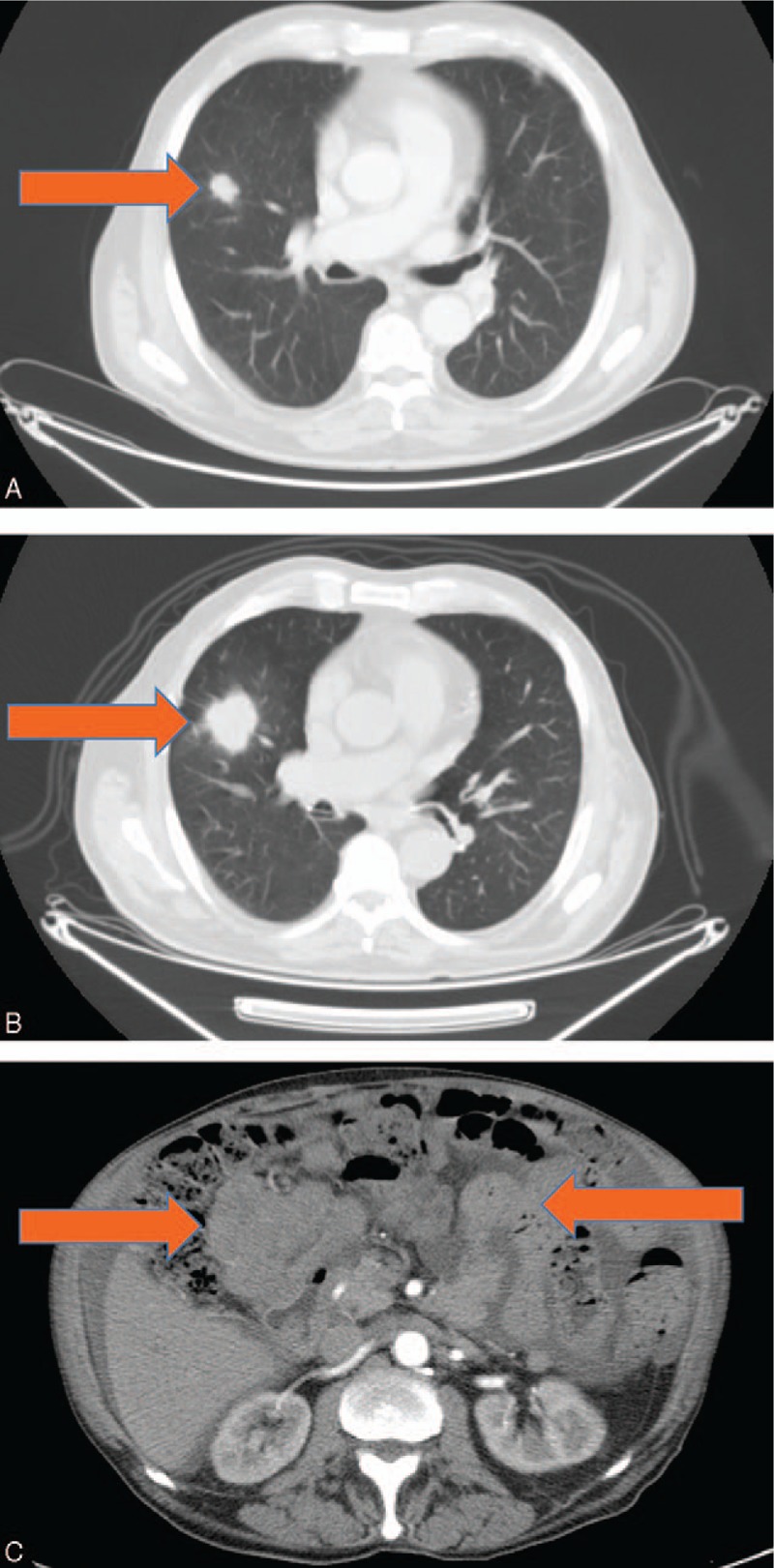
Relevant figures from CT-scans collected throughout the patient's clinical history. The arrows indicate lesions of interest. Notably, as the patient could not provide CT-scans performed before April 2009 in a different Institution, pictures of the lung adenocarcinoma located in the left lower lobe are not available. (A) CT-scan picture showing the SCC located in the right upper lobe in September 2010, before being treated with carboplatin-based chemotherapy; (B) CT-scan picture showing the same tumor (SCC) as in November 2011, progressing after carboplatin-based chemotherapy and periodical follow-up; (C) CT-scan picture showing diffuse abdominal lesions of PM.

The indeterminate nodule in the right upper lobe remained stable until September 2010, when an increase of its maximum diameters from 22 to 33 mm was reported; a positron emission tomography (PET) scan showed fluorodeoxyglucose (FDG) uptake limited to the right upper lobe lesion (SUV max: 6.2). Following this finding, the lesion was considered a metastasis of the original ADC and subsequently the patient received chemotherapy with carboplatin (AUC2), which was discontinued after 2 cycles for thrombocytopenia. During the subsequent assessments, the pulmonary lesion was substantially stable until November 2011, when a significant dimensional increase was observed. A subsequent PET-scan confirmed the right lung lesion as the only clearly detectable active site of disease (SUV max: 12.6, increased from the previous examination), while no distant metastases were identified; therefore, surgery with potential curative intent for oligo-metastatic disease was proposed. Hence, in January 2012, the patient underwent right upper lobectomy and radical lymphadenectomy with postoperative diagnosis of keratinizing and moderately differentiated squamous cell carcinoma (SCC) of the lung with positivity for p63 at IHC (pT2a G2 pN0 Mx, stage IB) (Fig. 2B). Although the clinical presentation could initially suggest a possible correlation between the 2 lung lesions, the IHC led to define 2 histologically distinct primary lung tumors. After surgery, the patient did not receive further treatments. In February 2014, metabolically active gastric lymphadenopathies and ascites were detected during follow-up, although no suspicious lesions were identified with esophagogastroduodenoscopy. Between October 2014 and January 2015, diffuse nodulations within the abdomen, morphologically compatible with peritoneal carcinomatosis, and a new lesion in the middle lobe of the right lung were identified. In February 2015, the patient was referred to our institution (Lung Cancer Unit; IRCCS AOU San Martino - IST, Genova, Italy), wherein he underwent biopsy of an easily accessible abdominal lesion located at the level of the right iliac fossa. At microscopic examination, the specimen was consistent with several small fibrous fragments diffusely infiltrated by an epitheliomorphic neoplasm composed of atypical cells, ranging from middle to large dimension, with well-represented eosinophilic cytoplasm, sometimes microvacuolated, and large nuclei, with prominent eosinophilic nucleoli; rare “hobnail cells” were identified and the neoplastic elements were arranged in solid nests, ribbons, and papillary structures. At IHC, expression of CK7, CK5&6, calretinin, and WT-1 was detected in neoplastic cells, whereas no expression of CK20, p63, MOC-31, TTF-1, and napsin-A was reported (Fig. 2C–H). On the basis of the morphology and the IHC pattern, the diagnosis of epithelioid PM was posed and, subsequently, the patient received chemotherapy with pemetrexed (500 mg/m^2^), which was discontinued after 2 cycles due to poor tolerance. Then, the patient experienced progressive worsening of clinical conditions and died in March 2015. Relevant images from CT-scans collected throughout the clinical history of the patients have been reported in Fig. 3.

In order to understand whether ADC, SCC, and PM were unrelated cancers or shared a common clonal evolution, WES analysis was performed on the 3 tumors by HiSeq 2500 sequencer (Illumina Inc, San Diego, CA, USA) as already described.^[12]^ Simultaneously, the WES of germinal gDNA obtained from peripheral blood was performed to subtract the germline background for the identification of somatic variants (see text, Supplemental Content 1, which illustrates samples processing and WES analysis).^[12–15]^ For this analysis, the ADC and the SCC samples were collected from stored surgical specimens (acquired during potentially curative surgery), while the PM sample derived from the tissue collected during the abdominal biopsy.

**Figure 4:**
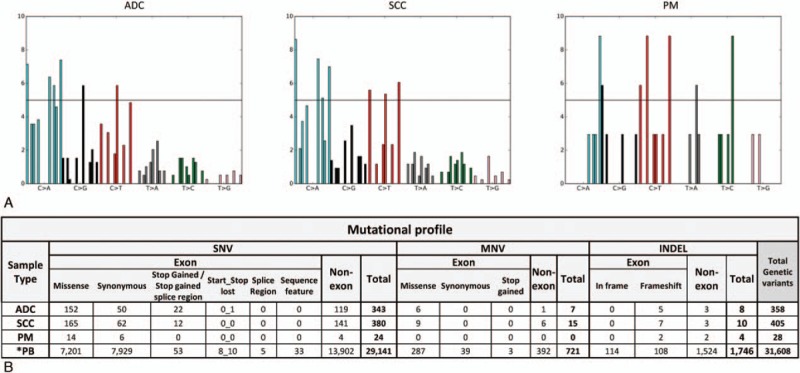
(A) Specific mutational signature for ADC, SCC, and PM according to the base substitutions.^[8]^ The substitution types are showed on the horizontal axis, whereas the percentages of base substitutions are displayed on the vertical axis. (B) Mutational profile of somatic (ADC, SCC, and PM) and germline (PB) gene variants divided in single nucleotide variant (SNV), multiple nucleotide variant (MNV), and INDEL. Each type of mutation was subdivided into exon or non-exon (intergenic regions, downstream and upstream regions, 5′UTR/3′UTR regions, splice regions, and intron regions) variants. “Stop gained”: variant causes a stop codon; “Start_Stop lost”: variant causes start codon to be mutated into a nonstart codon or variant causes stop codon to be mutated into a nonstop codon, respectively; “Splice region variant”: variant affective putative (Lariat) branch point from U12 splicing machinery, located in the intron; “Sequence Feature”: unknown/any extent of continuous biological sequence.

We firstly extracted the somatic mutational signature from all the tumors according to base substitutions, as already described by Alexandrov et al.^[8]^ This analysis displayed a predominance of C>A transversions in both lung cancers (ADC and SCC) (Fig. 4A), corresponding to a specific cancer signature related to tobacco consumption.^[8]^ In contrast, the PM did not exhibit any specific mutational signature, probably as a consequence of the few observed somatic variants (Fig. 4A). Then, we found that each tumor reported a specific set of somatic variants (358, 405, 28 in ADC, SCC, and PM, respectively; Fig. 4B; See Table, Supplemental Content 2A, Supplemental Content 2B, and Supplemental Content 2C, which list all somatic mutations found in ADC, SCC, and PM, respectively), which were not shared across the 3 tumors. Both ADC and SCC showed lung tumor hotspot mutations reported in the Catalogue of Somatic Mutations in Cancer (COSMIC; http://cancer.sanger.ac.uk/cosmic) database and described in lung cancers: *EHHADH* (COSM5247826), *KRAS* (COSM512), *OR4K2* (COSM1515038), and *TP53* (COSM6549) in ADC; *KIAA1324L* (COSM396629), *NFE2L2* (COSM396629), *PEG3* (COSM5284477), *POM121L12* (COSM393793), and *WAC* (COSM5311283) in SCC. Moreover, both histotypes carried mutations associated with potential therapeutic targets (*FLT3* and *HGF* in ADC; *MTOR* in SCC), or in a predictor of resistance to EGFR tyrosine kinase inhibitors (*KRAS* in ADC).

The enrichment analysis using Reactome 2015 (http://amp.pharm.mssm.edu/Enrichr/) also showed that different pathways were deregulated in ADC and SCC. Specifically, ADC was enriched with altered genes belonging to the MAPK pathway (p.Gly12Phe *KRAS*; c.∗76delC *MAP2K*; c.∗30C>T *MAP3K4*), whereas the mutations observed in SCC mostly affected genes involved in collagen modification, in extracellular matrix organization (p.His1331Gln *ADAMTS3*; p.Phe486Ser *COL19A1*; p.Ala75fs *LOX*; c.93 + 567C>A *SPP1*; p.Pro947Ser *LAMB1*; p.Met688Ile *A2M*), and in the meiotic synapsis pathway (p.Ser1801Gly *ATR*; p.Gln1747Glu *DIDO1*; c.1961 + 53A>T *SUN1*; c.17542-41A>C *SYNE1*). Conversely, the PM did not display COSMIC mutations or pathways associated with the carcinogenesis, probably due to the low number of somatic mutations (28); however, among these mutations, we identified 3 novel variants including 2 frameshift variants (p.Glu673fs *BAP1*; p.Glu1595fs *SETD2*) and a missense variant (p.Ser71Phe *WT1*).

Germline analysis was also performed in order to discover genetic variants potentially linked to cancer predisposition. Germinal gDNA sequencing identified a total of 31,608 genetic variants of which 15,790 and 15,818 occurred in exons and nonexons regions, respectively (Fig. 4B). In particular, 49% (7784/15,790) of the exon variants showed a high/moderate effect on the protein, whereas the 66% (10,397/15,818) of nonexon variants potentially modified the protein regulation based on effect prediction of SnpEff tool (http://snpeff.sourceforge.net).

As pathway analysis did not disclose enrichment pathways linked to tumor susceptibility, we focused on genes related to DNA repair or associated with cancer predisposition. The analysis identified 74 genetic variants in 59 genes related to DNA repair/cancer predisposition. Specifically, 21 out of 74 genetic variants have already been described to confer a high risk of cancer development and 7 of them were homozygous (rs3760413, *EME1*; rs26279, *MSH3*; rs8305, *POLI*; rs373572, *RAD18*; rs462779, *REV3L*; rs25487, *XRCC1;* rs1143634, *IL1B*) (Table 1). Finally, we found 5 single nucleotide polymorphisms (SNPs) (rs1948, *CHRNB4*; rs1051730, *CHRNA3*; rs16969968, *CHRNA5*; rs4950, *CHRNB3*; rs5320, *DBH*) involved in the etiology of the nicotine dependence (Table 1).

**Table 1:**
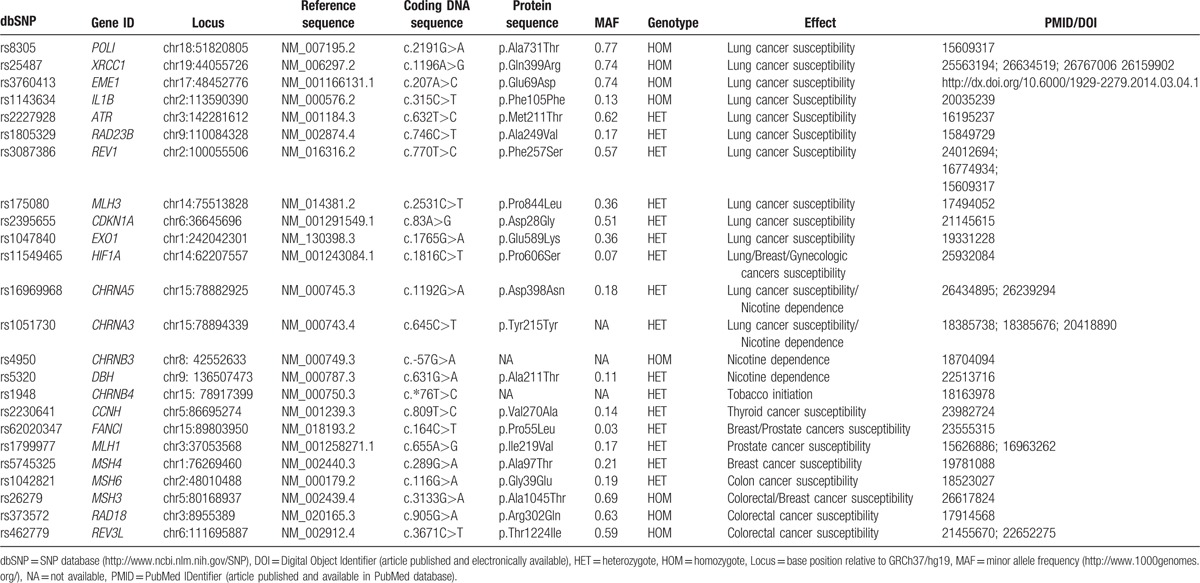
Single nucleotide polymorphisms associated with tumor susceptibility and nicotine dependence.

## 3 Discussion

Here, we describe an infrequent case of a patient who developed 2 histological distinct intrapulmonary tumors and a PM after 6 years. WES of the 3 tumors was performed to establish a clonal relationship. Although both lung ADC and SCC showed a similar mutational signature, characterized by a prominence of C>A substitutions, they did not share common somatic variants. Interestingly, the signature characterized by C>A mutations has been associated with smoke exposure in several cancers including lung ADC and SCC^[8]^; indeed, cigarettes contain a complex mixture of carcinogenic agents and these compounds could interact with DNA leading to the accumulation of somatic mutations. Recently, Warth et al^[6]^ analyzed a set of synchronous primary lung tumors demonstrating that clonally independent ADC and SCC tumors were mainly identified in heavy smoker patients. These data support the association between extensive smoking and the development of the 2 clonally unrelated lung tumors occurred in our case. Across 358 altered genes in the ADC, we found 6 (*KRAS, MAP2K1, MGAM, NF1, PPP3CA*, and *TP53*) of 38 genes significantly mutated in a cohort of 660 lung ADC.^[16]^ Of note, mutation in *PPP3CA* co-occurred with an activating *KRAS* mutation (COSM512) as already described by Campbell et al.^[16]^ In addition, the mutation in the *MGAM* gene has been also observed in a comprehensive genome-wide characterization by Cancer Genome Atlas Research Network among 18 genes found significantly mutated in 230 lung ADC tumors.^[17]^ Across the 405 SCC-mutated genes, we found only 1 gene (*NFE2L2*) of 20 genes recurrent mutated in 484 lung SCC tumors^[16]^; moreover, mutations in *NFE3L2* gene have also been identified in 34% of 178 lung SCC tumors profiled by Cancer Genome Atlas Research Networt.^[18]^

Furthermore, both lung tumors showed a specific gene signature linked to distinct pathways of activation. Specifically, the ADC harbored mutations in genes involved in EGFR signaling pathway, such as 2 novel genetic variants in the 3’UTR regions of *MAP3K4* and *MAP2K1* genes, and a hotspot mutation in the *KRAS* codon 12; as it is known, the EGFR signaling pathway is one of the most frequently altered pathways in this histology.^[19]^ On the contrary, the SCC carried several mutations in genes involved in the extracellular matrix organization, a pathway often deregulated in cancer.^[20]^ In particular, we found a novel frameshift deletion (c.221delC; p.Ala75fs) leading to a potential LOX protein destruction. *LOX* downmodulation has been found in SCC and its lack has been shown to induce the extracellular matrix disorganization leading to tumor development.^[21]^ Furthermore, in addition to being potentially involved in tumor development, some of the affected genes that were observed in this case might also play a relevant role in a targeted therapy approach in patients affected by lung cancer, possibly reducing sensitivity to currently registered agents or eventually representing potential targets for drugs that might become available for lung cancer in future. Although it is still unclear whether *KRAS* mutations are actually associated with resistance to EGFR inhibitors in lung cancer,^[22]^ aberrations of HGF signal are apparently involved in resistance to anti-EGFR and anti-VEGF targeted therapies.^[23]^ Contrarily, FLT-3 and mTOR might represent potentially actionable targets, as the former is sensitive to drugs such as dovitinib, while the latter is sensitive to everolimus.^[24]^

Conversely, in PM, the distribution of base substitutions did not match any specific mutational signature, probably as a consequence of a relatively limited number of observed mutations (28 variants in PM vs >350 in the lung cancer lesions). Peritoneal mesothelioma is an extremely rare tumor and our sequencing data were in accordance with a previous study in which the authors performed WES on 7 PM finding a low mutational rate and *BAP1* as the most altered gene.^[25]^ We also found an insertion in *BAP1*, potentially associated with a loss-of-function, and a deletion changing the reading frame in *SETD2,* a gene found altered in malignant pleural mesothelioma.^[26]^ In addition, we detected a novel mutation in the *WT1* transactivation domain (NM_000378.4; c.212C>T; p.Ser71Phe). Mutated *WT1* has been already described in mesothelioma; interestingly, Park et al^[27]^ reported a patient with PM that harbored a point mutation within the transactivation domain of *WT1* gene, demonstrating that this variant conferred an activation of its transcriptional role. However, the authors did not find any *WT1* mutations in a further set of 32 asbestos-related mesothelioma patients, thus concluding that the WT1 pathway could be involved in the malignant transformation of nonasbestos-related mesothelioma. These data suggest that the p.Ser71Phe *WT1* mutation might be implicated in the PM carcinogenesis process through the WT1 downstream pathway activation. Indeed, the mutation serine-71-phenylalanine (p.Ser71Phe) in *WT1* gene is a nonconservative mutation that alters the properties of the protein by replacing the small and polar serine with the large and bulky side chain of a phenylalanine.

According to the previous data and excluding a common lineage across the 3 tumors, we hypothesized that this patient could have an intrinsic predisposition to develop MPT. Indeed, the germinal gDNA sequencing showed that more than half of the variants were potentially associated with protein alterations. Notably, the analysis identified 21 genetic variants that were already described; of these, 62% were related to increased lung cancer risk. Among such variants, the association of the p.Glu589Lys in *EXO1* gene (rs1047840) with cigarette smoking has been described as conferring a significantly increased lung cancer risk, with a reported odds ratio equal to 1.72.^[28]^

To the best of our knowledge, this is the first study that investigates the whole exome mutational profile of 3 MPT aimed at defining the clonal origin of the tumor lesions and also the germline assets in order to discover an individual genetic susceptibility to cancer predisposition. Our data support the hypothesis that the development of the 3 tumors was clonally independent, as they do not share a common mutational profile; however, we could not exclude the presence of mutations in regulatory regions, omitted by WES. The patient also carried several SNPs involved in nicotine dependence and DNA repair. The carcinogenic effects of tobacco smoke together with both a DNA repair deficiency and the advanced age of the patient may have led to a high mutation rate in the lung cancer lesions. It is also known that chemotherapy might affect the mutational status of eukaryote cells.^[29]^ Despite the only 2 cycles of carboplatin, considering the interval between treatment and SCC tumor collection (about 14 months), we cannot exclude the mutagenic effect induced by carboplatin.

On the contrary, the low number of somatic mutations in PM suggests that its development is mainly caused by onset of mutations in driver genes (*BAP1* and *SETD2*) and that other mechanisms, such as microRNA deregulation, might be involved.^[30]^ In addition, the novel missense mutation in *WT1* gene may also explain the PM development regardless of asbestos exposure.

In conclusion, this study underlines how the germline assets could influence the cancer predisposition and how future WES studies on patients with MPT should be directed toward the genetic variants identification leading to cancer susceptibility. Our findings highlight the power of WES analysis in screening the mutational landscapes of patient with MPT in order to define the clonal feature and identify novel potential molecular targets for treatment.

## Supplementary Material

Supplemental Digital Content

## Supplementary Material

Supplemental Digital Content

## Supplementary Material

Supplemental Digital Content

## Supplementary Material

Supplemental Digital Content
