# Supplementary material for: Whole exome sequencing of independent lung adenocarcinoma, lung squamous cell carcinoma, and malignant peritoneal mesothelioma: A case report
Source: Medicine (Baltimore). 2016 Dec 2;95(48):e5447. doi: 10.1097/MD.0000000000005447 (PMC5134773; doi:10.1097/MD.0000000000005447)
Supplement: Supplemental Digital Content [file md-95-e5447-s001.pdf]

Whole Exome Sequencing of Independent Lung Adenocarcinoma, Lung Squamous Cell Carcinoma and Malignant Peritoneal Mesothelioma: a Case Report.

Irene, Vanni & Simona, Coco
Lung Cancer Unit,
IRCCS AOU San Martino - IST Istituto Nazionale per la Ricerca sul Cancro,
Genova, Italy

Sample processing and WES analysis

Isolation of gDNA and quality control

ADC and SCC samples were collected from stored surgical specimens, while the PM sample was obtained during the abdominal biopsy. Each FFPE tumor block was revised by the pathologist to check the cell tumor content (>50%) and in the PM a manual microdissection has been performed to achieve an adequate tumor cell content. gDNA was extracted starting from two slides (10 µm) using GeneRead DNA FFPE Kit (Qiagen, Hilden, Germany) including also a step with the uracil-DNA glycosylase enzyme, to remove the cytosine deamination artifacts. Three ml of peripheral blood (PB) were collected in EDTA-coated tube at the time of diagnosis of PM, and gDNA was isolated from white blood cells using QIAamp DNA Blood Mini Kit (Qiagen). The gDNA concentration was assessed by Qubit® 2.0 Fluorometer (Invitrogen, Carlsbad, CA, USA) (Figure 1B). Moreover, a gDNA quality control step was performed for each sample by 2200 TapeStation Instrument (Agilent Technologies Santa Clara, CA, USA) using Genomic DNA Screen Tape (Agilent Technologies). The analysis reported variable degradation status by a numerical measure of gDNA quality (DNA Integrity Number (DIN) ranges from 10 for not degraded gDNA to 1 for highly fragmented gDNA) (Figure 1A-B-C). The Ethics Committee of the IRCCS AOU San Martino-IST, Genoa (Italy) stated that it is not necessary to obtain an approval for this single case. However the patient signed an informed consent to perform the molecular analysis.

Figure 1. gDNA quantity and quality control

A

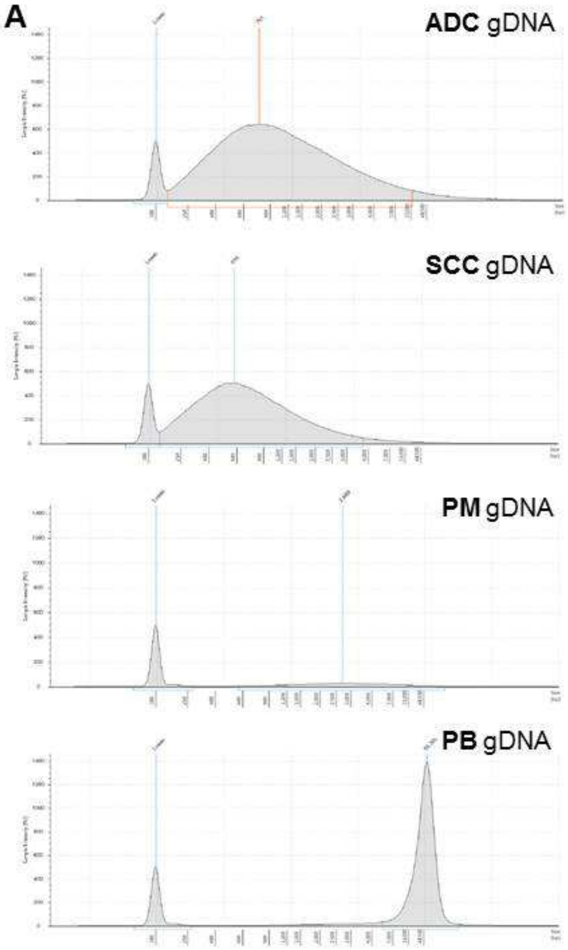

B

SAMPLE	DIN	[ ] TapeStation	[ ] QuBit
ADC	2.2	139.0 ng/ul	90.6 ng/ul
SCC	1.7	87.9 ng/ul	61.2 ng/ul
PM	4.9	6.91 ng/ul	6.0 ng/ul
PB	9.2	49.8 ng/ul	61.0 ng/ul

C

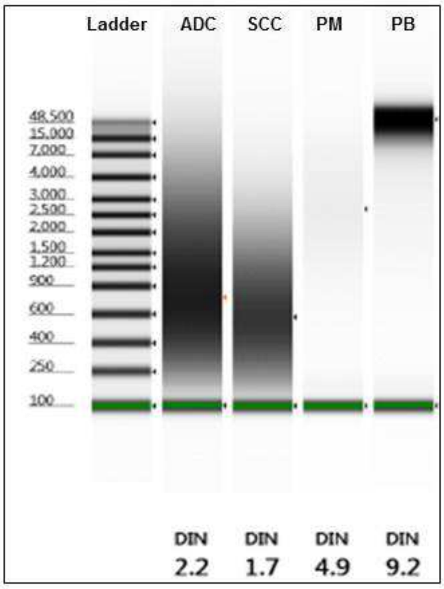

A) TapeStation profiles of gDNAs isolated from each tumor (ADC, SCC, PM) and PB; **B)** gDNA DIN, TapeStation and QuBit concentrations are displayed for each tumor (ADC, SCC, PM) and PB. **C)** Gel image obtained by TapeStation for each tumor (ADC, SCC, PM) and PB.

WES library preparation, hybridization capture and sequencing

Library preparation and sequencing were performed as described in Bonfiglio *et al.* 2016.[12] Specifically, 300 ng of gDNA samples extracted from ADC, SCC and peripheral blood, and 170 ng of gDNA extracted from PM were mechanically fragmented on an E220 focused ultrasonicator Covaris (Covaris, Woburn, MA, USA). Sheared gDNA was used to perform end repair, A-tailing and adapter ligation with Agilent SureSelect XT kit (Agilent Technologies), following the manufacturer instructions. Subsequently, the libraries were captured using Agilent SureSelect Human All Exon *v*5 (Agilent Technologies) probes, and finally amplified.

After quality control and quantification by Agilent 2100 Bioanalyzer (Agilent Technologies) and Qubit® 2.0 Fluorometer (Invitrogen, Carlsbad, CA, USA), the libraries were sequenced on an Illumina HiSeq 2500 platform (llumina Inc, San Diego, CA, USA) High Output mode, 2x100 cycles, with TruSeq SBS *v*3 chemistry.

The average target coverage achieved for tumor samples was 115X, 104X and 122X for ADC, SCC and PM respectively, and 40X for PB gDNA.

Bioinformatics analysis

The sequencing raw data were converted to fastq data by bcl2fastq software (Illumina) and then aligned to the human reference genome (hg19) using BWA-MEM aligner.[13] Duplicated reads were assessed by Picard MarkDuplicates and WES metrics were determined by Picard HsMetrics and Samtools.

Somatic variant calling

Somatic variant calling was performed with FreeBayes 1.0.2, using the following parameters: *--F 0.05 --C 2 --q 20 --pooled-continuous.*

After variant calling, the somatic variants were identified by applying a somatic filter described in the SpeedSeq pipeline.[14]

In order to avoid artifacts, the somatic variant list was further filtered in order to select variants having a variant quality (QUAL) higher than 20; at least one alternate observation on the forward strand (SAF>0) and one on the reverse strand (SAR>0); more than one read supporting the alternate balanced to both the left (5') (RPL) and the right (3') (RPR) of the alternate allele. Finally, only the variants covered at least 20X in all samples were considered for further analysis. For each tumor, the variant allele frequency of the specific set of somatic variants is 0% in the other two tumors and ≤5% in the normal sample.

Germline variant calling

Germline variant calling was performed with FreeBayes 1.0.2, using the following parameters: *--F 0.2 --C 2 --q 20.*

In order to avoid artifacts, the germline variant list was further filtered in order to select variants having a variant quality (QUAL) threshold higher than 20; at least one alternate observation on the forward strand (SAF>0) and one on the reverse strand (SAR>0); more than one read supporting the alternate balanced to both the left (5') (RPL) and the right (3') (RPR) of the alternate allele. Finally, only the variants covered at least 20X in all samples were considered for further analysis.

Variant annotation

Variant annotation and effect prediction were assessed by SnpEff,[15] and the following databases were used for annotation: dbSNP 146, COSMIC *v*75 and dbNSFP *v*2.9.

Mutational Signature Analysis

The mutational signature analysis was performed as described in Alexandrov et al using custom scripts.[8]

Validation of candidate gene variants

Nine selected candidate gene variants (*FLT3*, *TP53*, *HGF*, *ATR*, *MTOR*, *LAMB1*, *PEG3*, *WAC*, and *BAP1*), identified by WES (Table 1A), were validated by Sanger sequencing. A total of 30 ng of gDNA was amplified using specific primers according to the PCR conditions reported in Table 1B. Sequencing reactions were carried out using the Big Dye Terminator *v*3.1 kit (Thermo Fisher Scientific, Wilmington, DE, USA) and electrophoresis was run on 3130xl Genetic Analyzer (Thermo Fisher Scientific). Sequencing outputs were analyzed with Chromas Lite (Technelysium Pty., Australia).

WT1 gene variant was confirmed by Pyrosequencing (Table 1A). A total of 50 ng of gDNA was amplified using specific primers according to the PCR condition reported in Table 1B. The pyrosequencing assay was performed with a PSQ 524 96MA instrument (Qiagen); the sequencing reaction was performed with the Pyro Gold 525 reagent kit PSQ 96MA, according to the manufacturer instructions and the sequencing analysis conducted with the PSQTM 96MA software (v.2.02). The sequence to analyze for *WT1* variant detection was CTYCGACGTG CGGGACCTGA ACGCGCTG.

Table 1A. List of candidate genetic variants confirmed by Sanger Sequencing or Pyrosequencing

Gene	Locus	Coding DNA	Protein	Tumor
FLT3	chr13:28631588	c.380C>G	p.Ser127Cys	ADC
HGF	chr7:81335028	c.1798_1799delCCinsAG	p.Pro600Ser	ADC
TP53	chr17: 7577538	c.743G>T	p.Arg248Leu	ADC
ATR	chr3: 142217596	c.5401A>G	p.Ser1801Gly	SCC
MTOR	chr1: 11175490	c.7052G>T	p.Gly2351Val	SCC
LAMB1	chr7: 107595999	c.2839C>T	p.Pro947Ser	SCC
PEG3	chr19: 57326479	c.3331G>T	p.Asp1111Tyr	SCC
WAC	chr10: 28899726	c.1264C>G	p.Gln422Glu	SCC
BAP1	chr3:52436657	c.2015_2016insT	p.Glu673fs	PM
WT1	chr11:32456680	c.212C>T	p.Ser71Phe	PM

Table1B. Primers and PCR conditions used to validate the candidate gene variants

Gene	Technique	Forward primer sequence 5’ -3’	Reverse primer sequence 5’ -3’	Sequencing primer sequence 5’ -3’	Ta	PCR size (bp)
FLT3	Sanger	ggaacgctggagtaccaaac	tctccagcttgggtttctgtc	ggaacgctggagtaccaaac	58°C	152
HGF	Sanger	tgtgttgaatccagttgagagt	agccataaacactgcaactgg	tgtgttgaatccagttgagagt	56°C	141
TP53	Sanger	aggttggctctgactgtacc	agaaatcggtaagaggtgggc	agaaatcggtaagaggtgggc	60°C	204
ATR	Sanger	acccagctaccgtgacattt	cctcgttggtaggagcctct	cctcgttggtaggagcctct	58°C	205
MTOR	Sanger	gagggctatcgtttgccaac	gttcacctcaaagcagtccc	gagggctatcgtttgccaac	58°C	186
LAMB1	Sanger	ctggttactatggcgacccc	cctcccaaagtatgcacacga	cctcccaaagtatgcacacga	60°C	183
PEG3	Sanger	aggctcagacatggaagacc	gtgtgtactcccgactgtca	gtgtgtactcccgactgtca	55°C	155
WAC	Sanger	ttcttactgctggaccatctgc	agtaagaataccttgtgtagagagc	ttcttactgctggaccatctgc	55°C	84
BAP1	Sanger	gggtgatttctccagttgcc	cagttaagacagcagcgcat	gggtgatttctccagttgcc	58°C	203
WT1	Pyrosequencing	aggccagcgctgaacgtc	Biotin gttcaggtcccgcacgtc	gccgcagcaaatggg	65°C	92

Sanger primers were designed using Primer3 (http://primer3.ut.ee/). The primer sets for Pyrosequencing assay, which includes a PCR primer pair and a sequencing primer, were designed with Pyrosequencing Assay Design software (Biotage, Uppsala, SW).
